# Supplementary material for: The Experimental and In Silico-Based Evaluation of NRF2 Modulators, Sulforaphane and Brusatol, on the Transcriptome of Immortalized Bovine Mammary Alveolar Cells
Source: Int J Mol Sci. 2024 Apr 12;25(8):4264. doi: 10.3390/ijms25084264 (PMC11049820; doi:10.3390/ijms25084264)

Contents

[SFN vs. CTR Upregulated DEG – Biological process 2](#_Toc158996853)

[SFN vs. CTR Upregulated DEG – Cellular component 3](#_Toc158996854)

[SFN vs. CTR Upregulated DEG – Molecular function 4](#_Toc158996855)

[SFN vs. CTR Downregulated DEG – Biological process 5](#_Toc158996856)

[SFN vs. CTR Downregulated DEG – Cellular component 6](#_Toc158996857)

[SFN vs. CTR Downregulated DEG – Molecular function 7](#_Toc158996858)

[BRU vs. CTR Upregulated DEG – Biological process 8](#_Toc158996859)

[BRU vs. CTR Upregulated DEG – Cellular component 9](#_Toc158996860)

[BRU vs. CTR Upregulated DEG – Molecular function 10](#_Toc158996861)

[BRU vs. CTR Downregulated DEG – Biological process 11](#_Toc158996862)

[BRU vs. CTR Downregulated DEG – Cellular component 12](#_Toc158996863)

[BRU vs. CTR Downregulated DEG – Molecular function 13](#_Toc158996864)

[SFN vs BRU Upregulated DEG – Biological process 14](#_Toc158996865)

[SFN vs BRU Upregulated DEG – Cellular component 15](#_Toc158996866)

[SFN vs BRU Upregulated DEG – Molecular function 16](#_Toc158996867)

[SFN vs BRU Downregulated DEG – Biological process 17](#_Toc158996868)

[SFN vs BRU Downregulated DEG – Cellular component 18](#_Toc158996869)

[SFN vs BRU Downregulated DEG – Molecular function 19](#_Toc158996870)

# SFN vs. CTR Upregulated DEG – Biological process


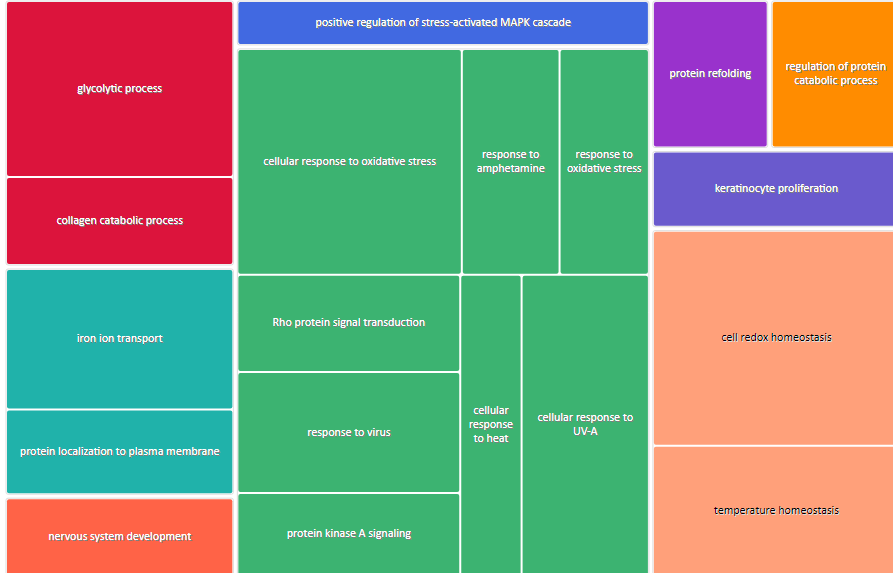


# SFN vs. CTR Upregulated DEG – Cellular component


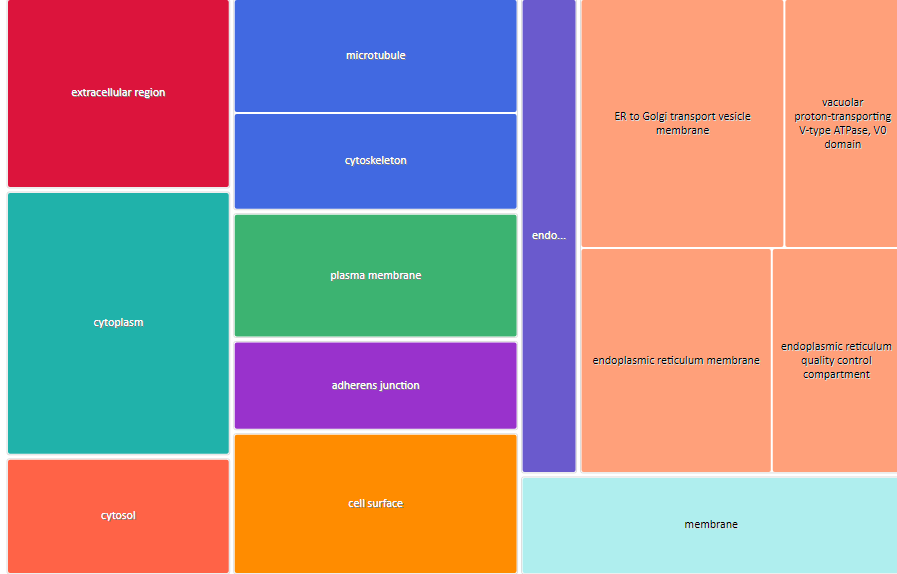


# SFN vs. CTR Upregulated DEG – Molecular function


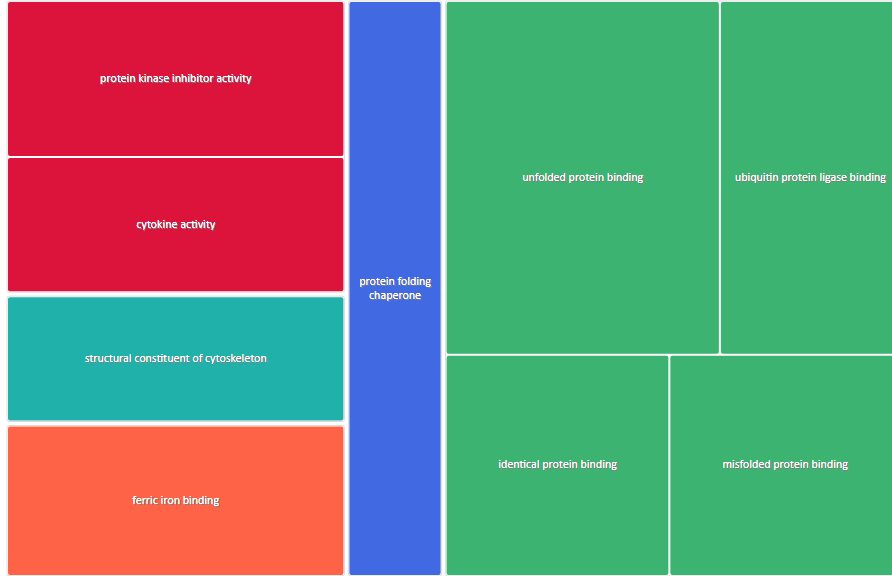


# SFN vs. CTR Downregulated DEG – Biological process


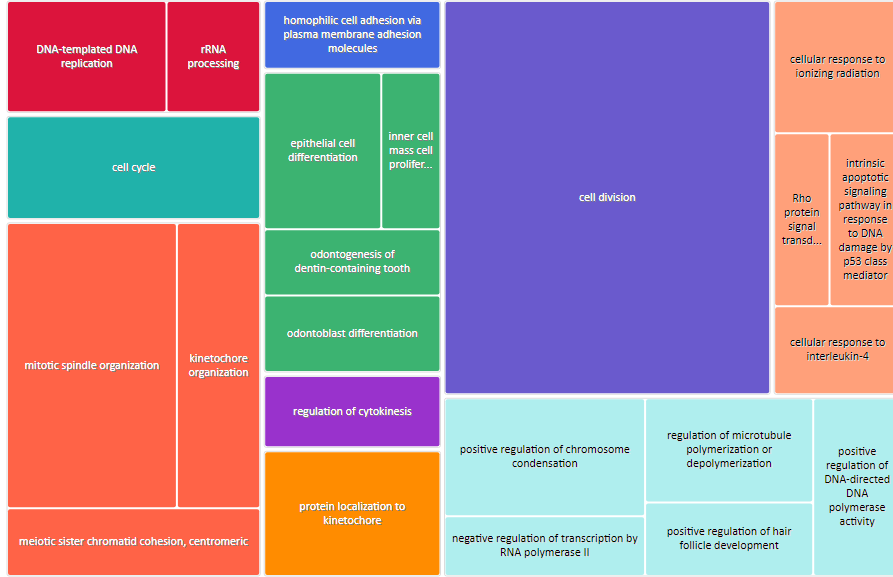


# SFN vs. CTR Downregulated DEG – Cellular component


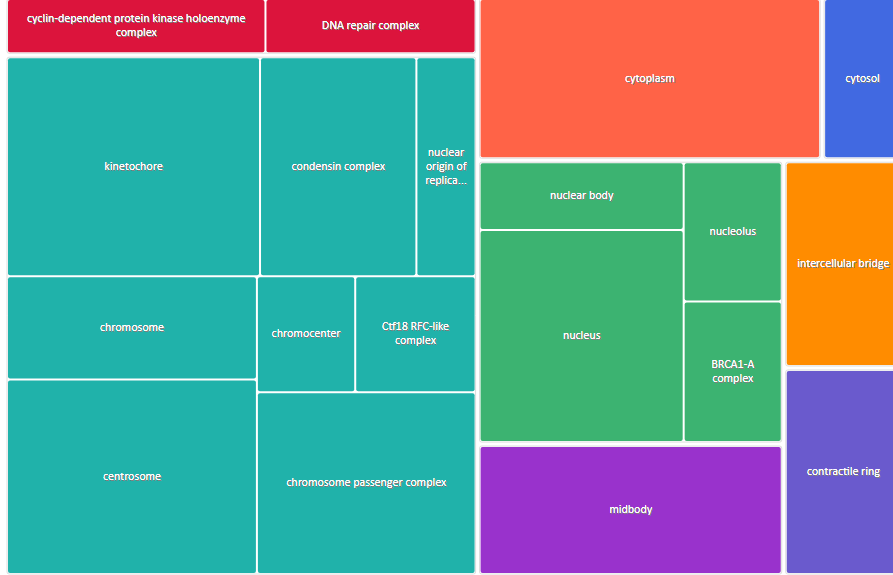


# SFN vs. CTR Downregulated DEG – Molecular function


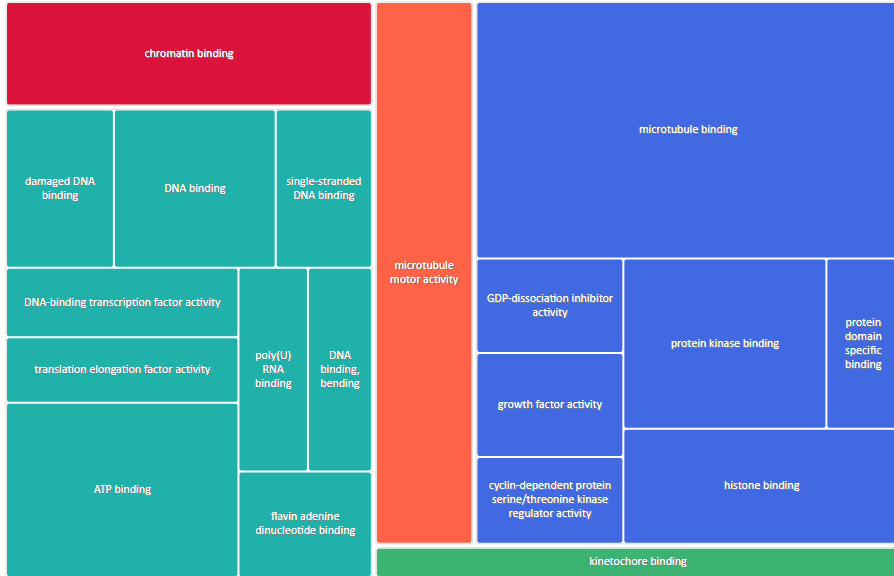


# BRU vs. CTR Upregulated DEG – Biological process


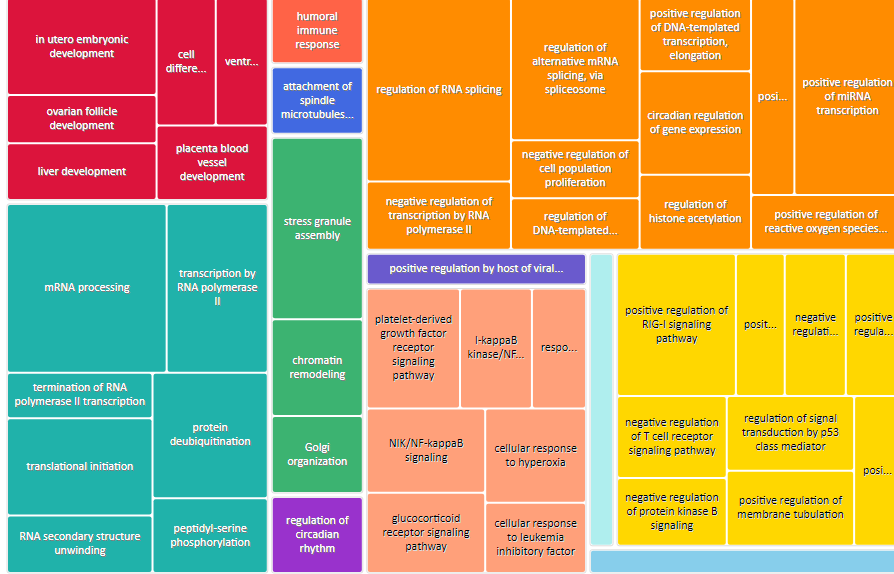


# BRU vs. CTR Upregulated DEG – Cellular component


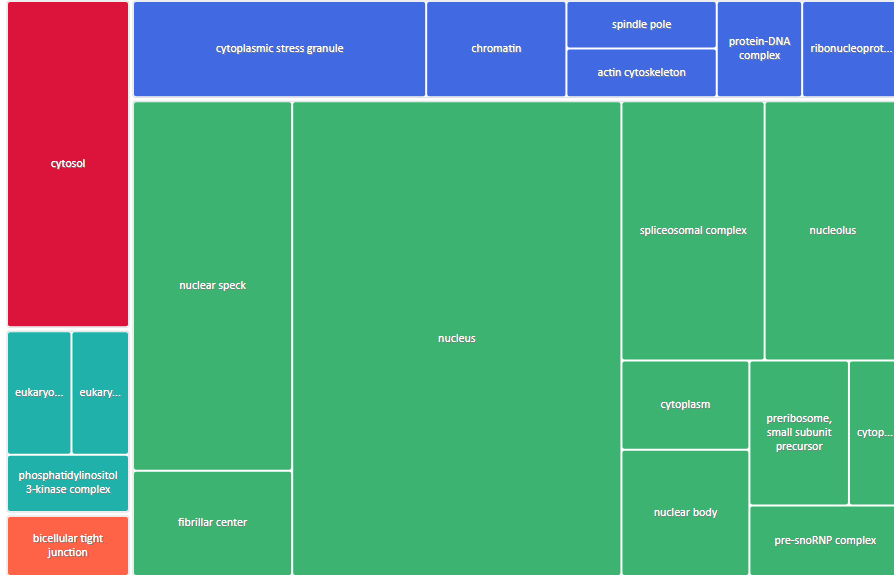


# BRU vs. CTR Upregulated DEG – Molecular function


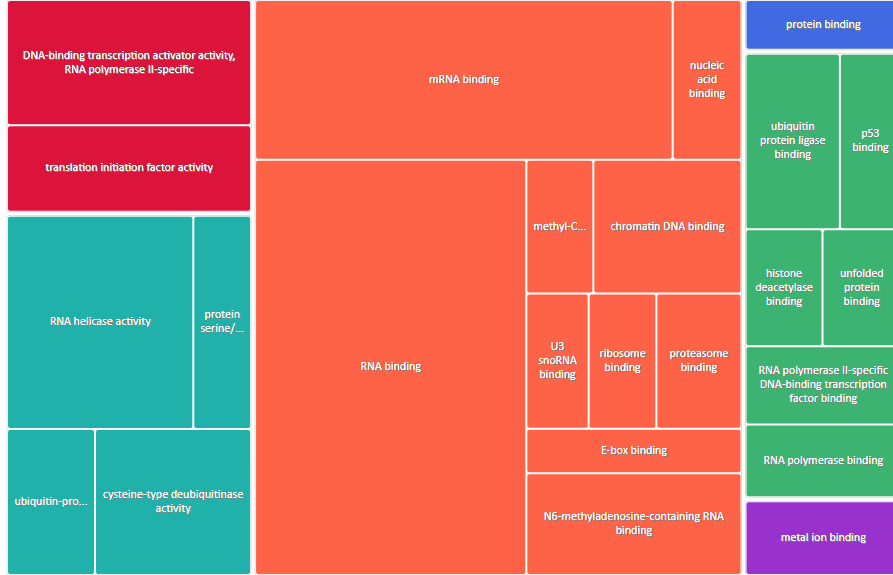


# BRU vs. CTR Downregulated DEG – Biological process


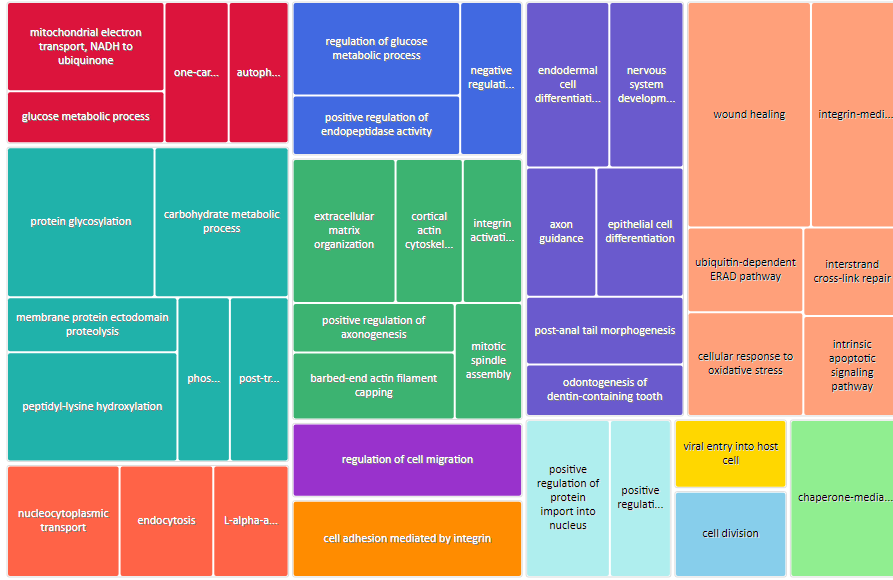


# BRU vs. CTR Downregulated DEG – Cellular component


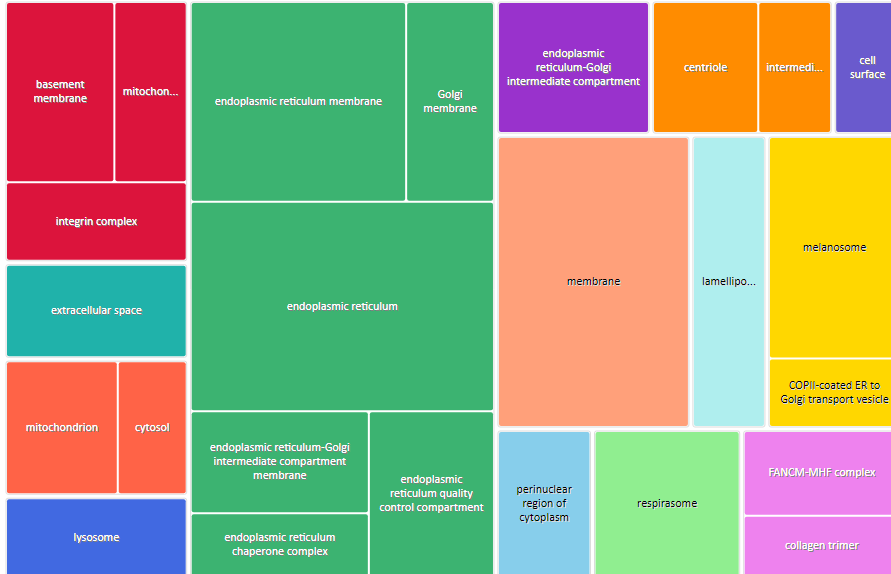


# BRU vs. CTR Downregulated DEG – Molecular function


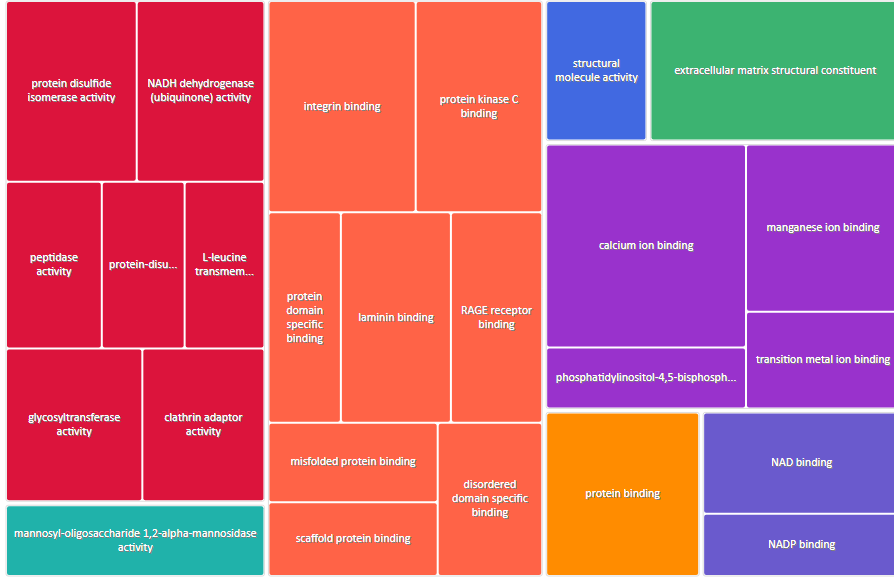


# SFN vs BRU Upregulated DEG – Biological process


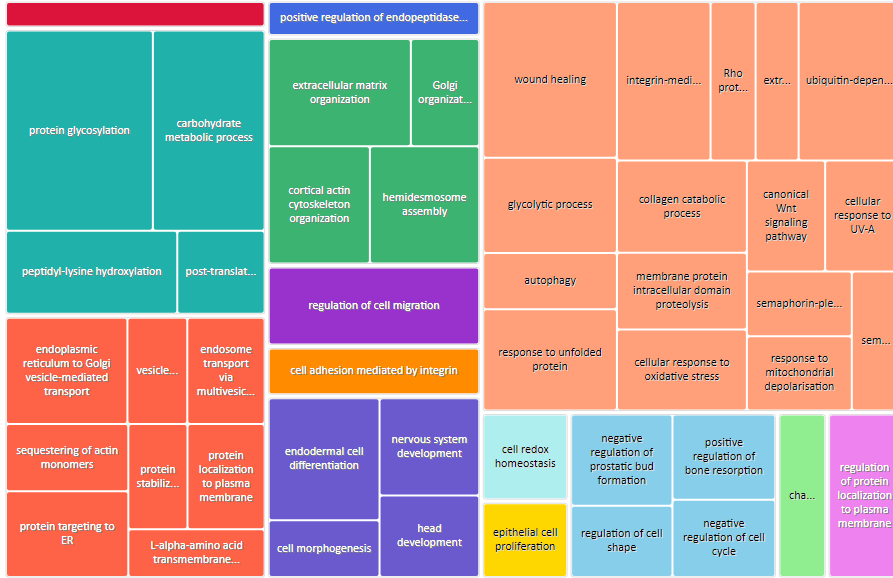


# SFN vs BRU Upregulated DEG – Cellular component


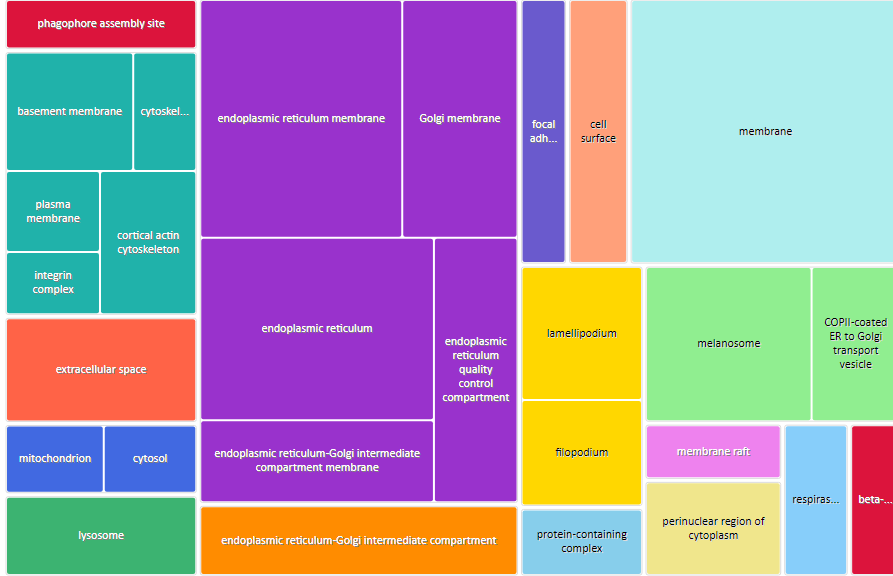


# SFN vs BRU Upregulated DEG – Molecular function


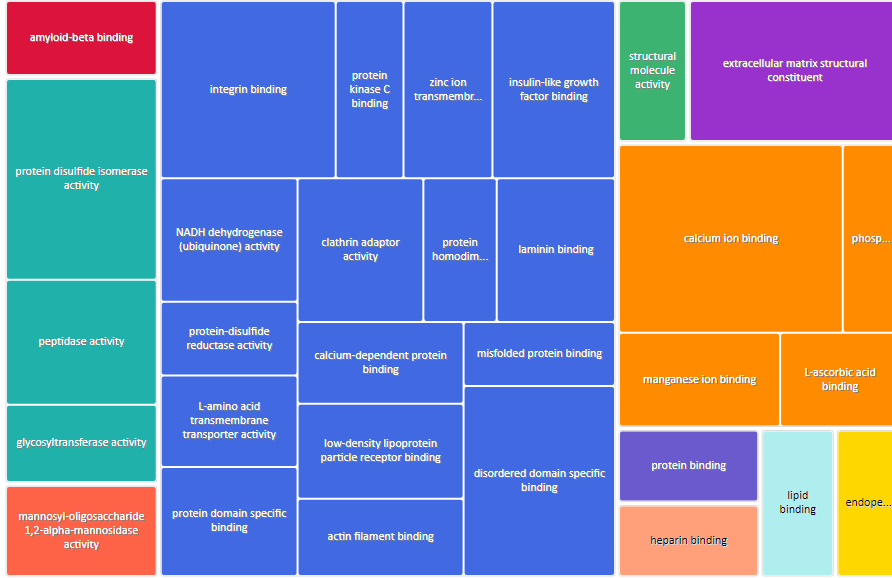


# SFN vs BRU Downregulated DEG – Biological process


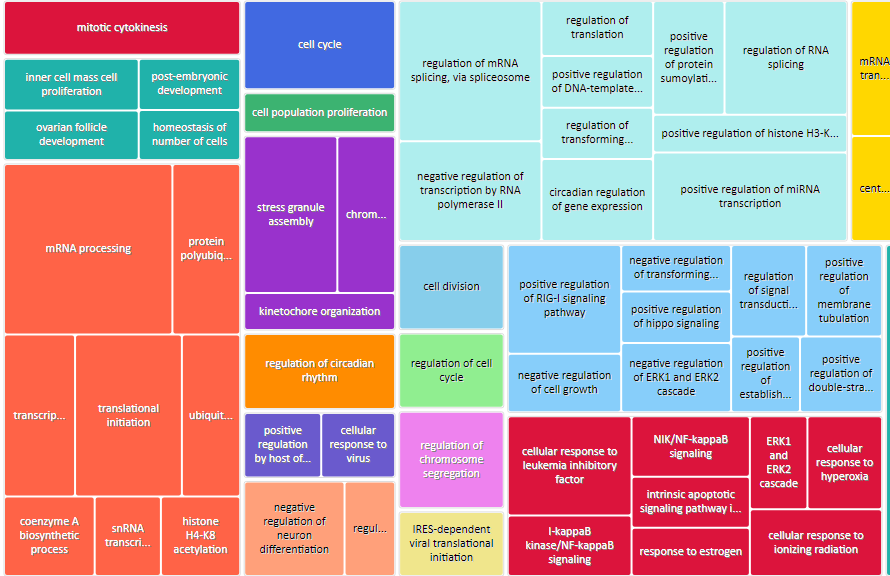


# SFN vs BRU Downregulated DEG – Cellular component


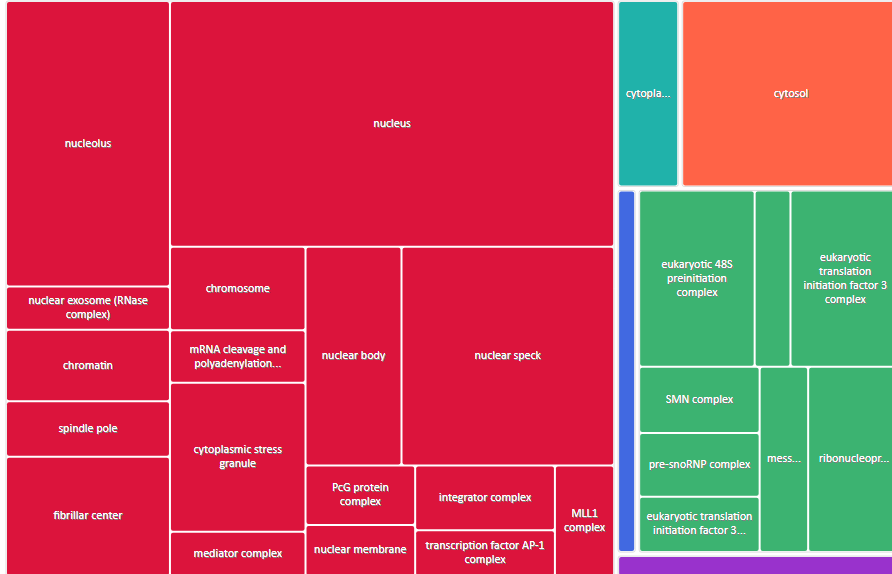


# SFN vs BRU Downregulated DEG – Molecular function


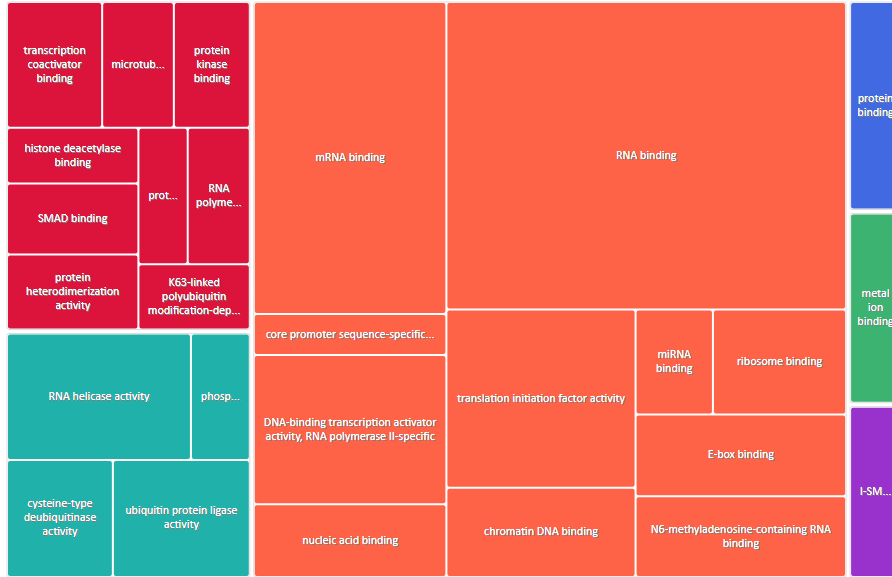

Supplement: Supplementary file 1 [file ijms-25-04264-s001.zip › Supplementary File S5.docx]
